# Detection of Anti-Counterfeiting Markers through Permittivity Maps Using a Micrometer Scale near Field Scanning Microwave Microscope

**DOI:** 10.3390/s21165463

**Published:** 2021-08-13

**Authors:** José D. Gutiérrez-Cano, José M. Catalá-Civera, Pedro J. Plaza-González, Felipe L. Peñaranda-Foix

**Affiliations:** Institute of Information and Communication Technologies (ITACA), Universitat Politècnica de València, Camino de Vera s/n, 46022 Valencia, Spain; jmcatala@dcom.upv.es (J.M.C.-C.); pedplago@itaca.upv.es (P.J.P.-G.); fpenaran@dcom.upv.es (F.L.P.-F.)

**Keywords:** document fraud, microwave imaging, near-field scanning microwave microscope, permittivity

## Abstract

This paper describes the use of microwave technology to identify anti-counterfeiting markers on banknotes. The proposed method is based on a robust near-field scanning microwave microscope specially developed to measure permittivity maps of heterogeneous paper specimens at the micrometer scale. The equipment has a built-in vector network analyzer to measure the reflection response of a near-field coaxial probe, which makes it a standalone and portable device. A new approach employing the information of a displacement laser and the cavity perturbation technique was used to determine the relationship between the dielectric properties of the specimens and the resonance response of the probe, avoiding the use of distance-following techniques. The accuracy of the dielectric measurements was evaluated through a comparative study with other well-established cavity methods, revealing uncertainties lower than 5%, very similar to the accuracy reported by other more sophisticated setups. The device was employed to determine the dielectric map of a watermark on a 20 EUR banknote. In addition, the penetration capabilities of microwave energy allowed for the detection of the watermark when concealed behind dielectric or metallic layers. This work demonstrates the benefits of this microwave technique as a novel method for identifying anti-counterfeiting features, which opens new perspectives with which to develop optically opaque markers only traceable through this microwave technique.

## 1. Introduction

Even though digitalization is changing today’s society, currency counterfeiting or document fraud are still serious threats that weaken economies and allow criminals to have a certain freedom of movement [1]. Anti-counterfeiting techniques are continuously being improved to incorporate innovative solutions to prevent document fraud, such as luminescent nanowire-based papers, invisible photoluminescent inks or chipless-RFID technologies [2,3,4].

Banknotes, for example, include a vast number of security features to prevent counterfeiting, consisting in the addition of markers that produce traceable properties in a certain range of the electromagnetic spectrum [5,6,7], such as watermarks, security threads, holograms, optically variable inks, micro lettering, intaglio printing, and infrared (IR), ultraviolet (UV), or magnetic (MG) marks. Among all these elements, watermarks are one of the most widely recognized security features by the common user. These marks are made by the deposition of cellulosic fibers with different densities during the production of the paper sheet, creating images that are visible when viewed against the light and are usually counterfeited by digital printing on the paper [8], which can be hard to detect by only visual means. Concerning the methods for identifying anti-counterfeiting features, watermarks, security threads, holograms, optically variable inks, and micro lettering are commonly detectable in the visible spectrum. Other security features generate a differential mark in the visible spectrum when illuminated with IR or UV light. These and other additional security features are typically employed by anti-counterfeiting machinery used in stores, ATMs, banks and for forensic verification through IR, UV, MG, or X-ray sensors. However, to the best of our knowledge, the microwave part of the electromagnetic spectrum has not been used before to detect any kind of security feature in document or banknote anti-counterfeiting applications.

Microwave spectrum refers to electromagnetic signals between 300 MHz and 300 GHz [9]. Microwave signals might outperform existing anti-counterfeiting technologies at different frequency ranges, since electromagnetic radiation at microwave frequencies shows certain penetrability in some materials, such as metals, dielectrics, or semiconductors, allowing for the development of optically opaque markers that are only traceable with microwave detectors.

Permittivity is a fundamental property of dielectric materials describing their interaction with electromagnetic fields [10] and is the most studied parameter at microwave frequencies. Permittivity studies have historically been linked to telecommunications systems [11]; nevertheless, microwave techniques for non-destructive testing and evaluation have also been frequently employed for a good range of industrial and scientific applications [12,13,14,15,16].

Permittivity measurement techniques have usually been designed to fit the specific size and shape of the samples under analysis [10]. For laminate and homogeneous dielectric surfaces, the preferred methodologies are based on stripline or microstripline resonators [17,18], split cylinder resonators [19,20,21], split-post dielectric resonators [22], or open-ended coupled coaxial probes [23]. However, for heterogeneous planar materials, near-field scanning microwave microscopes (NSMM) can provide dielectric property maps, directly related to the different components of the materials under test (MUT).

NSMM devices can measure the electromagnetic response of materials on a length scale significantly smaller than the wavelength of the emitted signal [24]. This involves a probe tip size (D) shorter than the probe-to-sample distance (r) and far shorter than the wavelength of the electromagnetic wave (λ) in order to ensure near-field radiation (D ≤ r << λ). Thus, the spatial resolution is given by D instead of λ.

The basic design of a typical NSMM comprises a near-field evanescent probe, a microwave source and detector (typically a vector network analyzer, or VNA), and a positioning (X–Y axis) stage to perform the pixel-by-pixel scanning of a sample of planar materials [25]. Even though there is a remarkable diversity of structures utilized as apertures for the evanescent probes, sharp coaxial tips are the most widely employed [24,25,26]. Two main detection modes are used depending on the probe configuration [24]. The non-resonant mode involves a transmission line section wherein detection is achieved by monitoring the electromagnetic wave reflected or transmitted from the sample. The resonant configuration, on the other hand, tracks the changes in the resonance parameters (resonant frequency and quality factor) caused by variations in the sample properties.

There is also diversity in the way that the measurable quantities—reflection, transmission, or resonance parameters—are related to the physical properties of the materials, including permittivity. These works include procedures such as lumped element models, full-wave analysis, or the cavity perturbation method (CPM) [27,28,29].

Recent developments in near-field scanning microwave microscopy have focused on increasing the special resolution to the nanoscale level [30]. To achieve such a high resolution, employing a distance-following technique is essential to keep the probe either in soft contact or at a distance of only a few nanometers from the sample [26]. Thus, NSMM devices with nanometer resolution require the support of a different nanoscale microscope technology, such as that of atomic force microscopes (AFM) or scanning tunneling microscopes (STM), to meet the distance-following needs. However, the detection of anti-counterfeiting features, especially watermarks, would require lower resolutions than nanoscale, and can benefit from a micrometer scale NSMM device with a much simpler and compact set-up, wherein permittivity measurements on larger non-homogeneous samples could be performed.

In this work, we describe the unprecedented use of microwave energy (technology) for the identification of markers on anti-counterfeiting applications. We employed a micrometer scale NSMM specially developed to detect the density variations of paper inside a banknote watermark through permittivity maps. In addition, the unique penetration capabilities of electromagnetic waves at microwave frequencies allowed the detection of new opaque markers hidden behind dielectric or metallic layers, only traceable with this microwave technique.

The developed NSMM device allows for the permittivity measurements of heterogeneous planar materials around the ISM microwave frequency of 2 GHz. An in-house vector network analyzer was embedded in the device to measure the response of the near-field probe, thereby avoiding the need for a full-featured VNA and making the instrument portable and easy to use. Unlike previous approaches, the permittivity calculation made use of a displacement laser and an enhanced CPM with calibration coefficients for different tip-to-sample distances, avoiding the need of distance-following techniques and increasing the robustness of the system.

Additionally, the detection capabilities of the proposed device can provide scientists and industrialist with a convenient tool for a wide range of sensing applications to analyze and characterize heterogeneous planar objects.

## 2. Design of the NSMM

### 2.1. The Near-Field Microwave Probe

**Figure 1:**
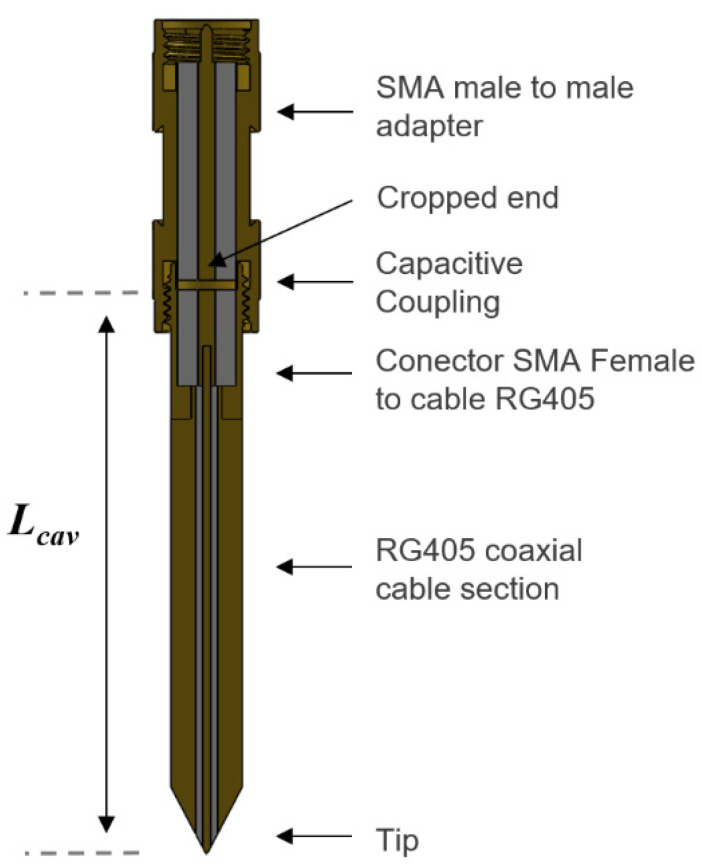
Geometry of the coaxial resonator implemented in the near-field microwave microscope, L_cav_ = 55.4 mm and R_tip_ ≈ 25 μm.

Figure 1 shows a drawing of the near-field evanescent probe implemented in the NSMM. The probe was designed as an open λ/2 coaxial resonator [26]. A section of a standard rigid RG405 coaxial cable was used to manufacture the central section of the resonator.

The near-field radiating end of the coaxial aperture was sharpened to increase the spatial resolution when positioned close to the MUT, around 25 µm [31]. An SMA female connector was fitted to the other end of the RG405 coaxial cable to connect the resonator to the microwave emitter and receiver. Taking this set-up into account, we fixed the length of the cavity to 55.4 mm for an operating frequency of around 1.85 GHz, choosing the first resonant frequency of the coaxial resonator.

The feeding network used to couple the microwave energy into the resonator was a capacitive coupling gap created by bringing the open end of the resonator close to another open-ended section, implemented by cutting the inner conductor of a male–male SMA adapter.

Finally, this SMA adapter was connected to the microwave analyzer that generates the microwave signals required by the evanescent probe to interact with the MUT and receive a response to be able to establish the resonator’s resonance parameters.

### 2.2. Single-Port Microwave Analyzer

**Figure 2:**
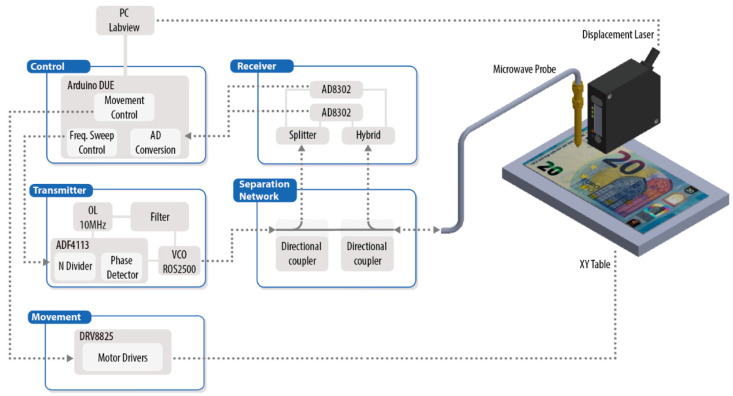
Schematic diagram of the in-house vector network analyzer for measuring the reflection (S_11_) of the near-field microwave microscope.

Figure 2 depicts a schematic diagram of the electronic module developed to operate the NSMM device. The generation and reception of microwave signals is comprised of four main subsystems: microwave transmitter, microwave receiver, separation network, and control unit.

The microwave transmitter was based on an ADF4113 (Analog Devices) frequency synthesizer, which, together with an external loop filter and a voltage-controlled oscillator (ROS-2500 Mini-Circuits, Brooklyn, NY, USA), formed a complete PLL able to run frequency sweeps around the resonance of the microwave microscope (2 GHz). The separation network included two directional couplers (BDCA 1-7-33+ from Mini-Circuits, Brooklyn, NY, USA) that were used to isolate the reference signal from the transmitter and the reflected signal from the near-field coaxial cavity.

These two signals were compared inside the receiver, which was based on an AD8302 RF/IF gain and phase detector (Analog Devices, Norwood, MA, USA) [32], providing analog outputs depending on the magnitude loss ratio and the phase difference between inputs. All the signals were converted to digital by a control unit based upon a microprocessor system and connected to a personal computer for further processing. Together, all these devices made up an in-house affordable single port VNA (reflectometer) similar to the system reported in [33].

In this new design, the PIC16C773 microcontroller of [33] was substituted by an Arduino DUE open-source electronics platform based on a 32-bit ARM core microcontroller. This platform allowed a convenient integration of the microwave devices and the mechanical X–Y positioning system, which thus reduced the latency times associated with the synchronizing of all the parts and increased the scanning speed.

The Arduino board and the LabVIEW-based software controlled the whole measurement process from the output magnitude and phase signals of the receiver to the calculating of the resonance parameters and dielectric properties, as well as displaying the results.

A standard calibration procedure with an OSL calibration kit (85052B Standard Mechanical Calibration Kit-3.5 mm, Keysight Technologies, Santa Rosa, CA, USA) was set from 1.8 to 1.85 GHz to cover the frequency response of the probe (see Section 3.1).

### 2.3. Positioning Stage

For the positioning subsystem, we used a commercial XY-stage (KT-70, proxxon) driven by two stepper motors and drivers (DRV8825, Texas Instruments, Dallas, TX, USA), which allowed for a pixel-by-pixel scanning with a maximum scan range of 150 mm × 70 mm and a resolution of 5 µm.

A vacuum table was attached to the XY-stage to fix the documents during the measurement procedure and avoid disturbances from folds in the documents or planar materials under test. The resonant probe was attached to the central axis of the XY-stage, employing a vertical, manually-driven micrometric positioner. A commercial laser displacement sensor (HL-G103-SJ, Panasonic, Osaka, Japan) with a resolution of 0.5 µm was placed close to the aperture of the microwave probe in order to precisely measure the tip-to-sample distance.

**Figure 3:**
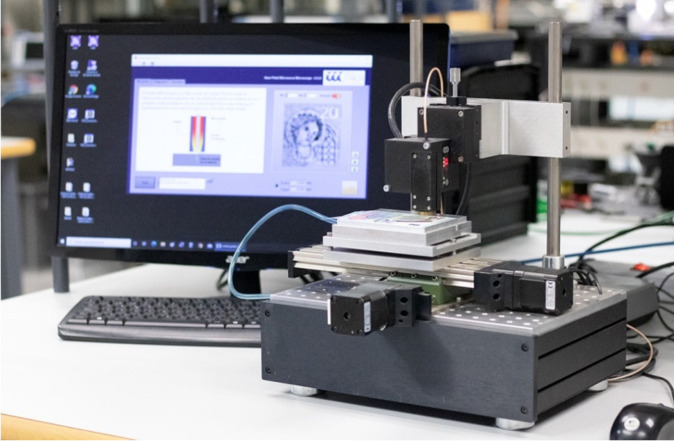
Picture of the near-field microwave microscope system.

The LabVIEW-based software was used to define the scanning parameters (area to scan, frequency sweep configuration…) and load them into the Arduino board. Next, the Arduino firmware autonomously controlled the scan process synchronized with the calculation of microwave parameters. The picture in Figure 3 shows the developed integrated NSMM system that operates as a standalone portable measurement instrument.

## 3. Permittivity Measurements with NSMM

### 3.1. Microwave Probe Response

**Figure 4:**
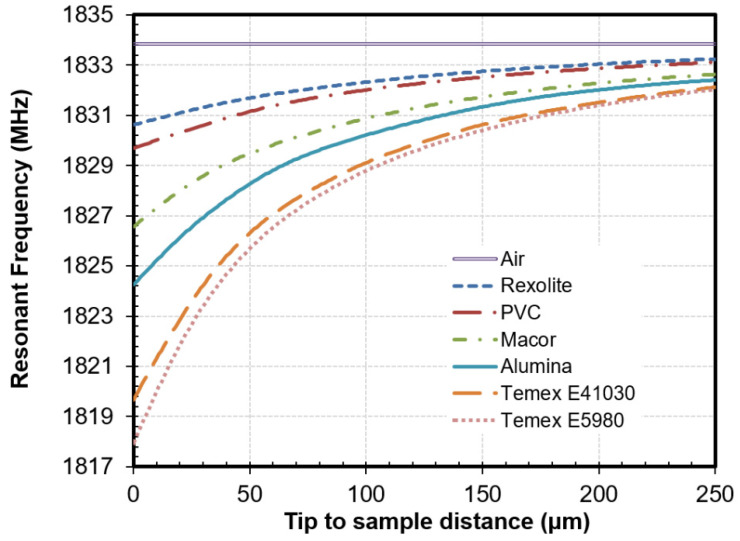
Variation of the resonant frequency as a function of the tip-to-sample distance for different reference materials.

The response of the near-field microwave resonator, described in the previous section, was assessed by measuring the resonant frequency (*f_r_*) and Q-factor (*Q*) parameters of some reference materials, covering a wide range of dielectric properties (see Table 1) as a function of the tip-to-sample distance. The size of the rod-shaped materials (15 mm high and with a diameter of 9.8 mm) was sufficient enough to be considered as infinitely wide and thick materials for the probe tip size and tip-to-sample distances [25,34]. The resonance parameters were determined from the reflection measurements by means of the linear fractional curve fitting procedure published by Kajfez [35,36]. Figure 4 shows the resonant frequency variation as a function of the tip-to-sample distance for the different materials measured. Because of the radiation characteristic of the open-ended coaxial probe tip, the quality factor calculated from the measurements exhibited small variations for the selected tip-to-sample distances.

The maximum frequency shift between the measurement of air and the material with the highest dielectric constant, Temex E5980 (67.39), occurred for the smaller tip-to-sample distance (see Figure 4). For the soft contact distance (0 μm), the maximum detected deviation was 16 MHz. From that point on, the maximum frequency shift decreased exponentially as the tip-to-sample distance increased.

Based on these results, the primary probe-to-sample distance was selected to be 100 μm, leaving the Z stage fixed during the rest of the measurements. At that tip-to-sample distance, the maximum frequency shift was 5 MHz. Even though this variation offered a good resolution throughout the complete permittivity range, the measurement resolution improved for those materials with lower dielectric constant values. For instance, at 100 μm, the frequency deviation exhibited between Rexolite and PVC, with a dielectric constant of 2.54 and 3.09 respectively, was 0.3 MHz, which is similar to the deviation reached between Temex E41030 and Temex E5980, with dielectric constant values of 28.28 and 67.25, respectively.

### 3.2. Cavity Perturbation Method (CPM)

Cavity perturbation method (CPM) was applied to determine the permittivity of the MUT from the measured resonance parameters. Since the earliest work on this procedure published by Bethe and Schwinger in 1943 [37], CPM has become one of the most widely employed techniques used to calculate dielectric properties using microwave resonators. The method assumes that the electromagnetic field of the resonant cavity is barely perturbed by placing the MUT in the structure. Under this premise, dielectric properties can be calculated using the relative shift of the resonance parameters with and without the MUT.

In an NSMM, the volume of the fields inside the cavity obviously outweighs their volume in the MUT and, thus, the CPM can also be employed to analyze a near-field cavity resonator [24]. Most of the CPM reported in the literature for NSMM structures made use of the theoretical approach proposed by Gao and Xiang [27], who studied the fields inside the sample, assuming an excitation illuminated by a spherical tip. They assumed an iterative image charge problem and solved the CPM equations to relate the *f_r_* and *Q* shifts to the permittivity of the MUT and other parameters depending on the tip-to-sample distance and the size of the probe. However, some authors have described issues in this model for longer tip-to-samples distances [34] and for some apertures that cannot be described with a spherical tip.

For the specific geometry of Figure 1, we assumed a quasi-static electric field inside the MUT [38] and related the permittivity of the MUT to the relative shift in the resonant frequency and the quality factor according to Khanna et al. [39], with the following formulas:

(1) $$\varepsilon^{\prime }=1+\frac{-\frac{\Delta f}{f}\left(\eta +N\frac{\Delta f}{f}\right)-N{\left[\Delta \left(\frac{1}{2Q}\right)\right]}^{2}}{{\left(\eta +N\frac{\Delta f}{f}\right)}^{2}+N^{2}{\left[\Delta \left(\frac{1}{2Q}\right)\right]}^{2}}$$

(2) $$\varepsilon^{\prime \prime }=\frac{\eta \Delta \left(\frac{1}{2Q}\right)}{{\left(\eta +N\frac{\Delta f}{f}\right)}^{2}+N^{2}{\left[\Delta \left(\frac{1}{2Q}\right)\right]}^{2}}$$

where *ε′* = dielectric constant (dimensionless); *ε″* = loss factor (dimensionless); *f* = resonant frequency (s^−1^); *Q* = quality factor (dimensionless); *η* = sample filling factor (dimensionless); *N* = sample depolarization factor (dimensionless). The relative shifts can be written as follows [40]:

(3) $$\frac{\Delta f}{f}=\frac{f_{s}-f_{0}}{f_{s}}$$

(4) $$\Delta \left(\frac{1}{2Q}\right)=\frac{f_{0}}{f_{s}}\cdot \frac{1}{2}\cdot \left(\frac{1}{Q_{s}}-\frac{1}{Q_{0}}\cdot \frac{f_{s}^{2}}{f_{0}^{2}}\right)$$

where *f*_0_ and *Q*_0_ are the resonance frequency and quality factor of the open-air cavity, respectively, and *f_s_* and *Q_s_* are the cavity’s parameters with a dielectric material placed near the tip of the probe.

For an infinite size MUT, the parameters *N* and *η* depend on several factors, such as the resonant mode, the geometry of the cavity, or the tip-to-sample distance, and are usually calibrated by measuring reference materials of known dielectric properties [41].

Three materials—air, Macor (*ε′* = 5.68), and Temex E5980 (*ε′* = 67.25)—were selected for calibrating the *N* and η parameters at a tip-to-sample distance of 100 μm. Nevertheless, the irregular height of the material, as well as defects in the horizontality of the base that holds the sample, may cause variations in the tip-to-samples distances around the established gap of 100 μm. For this reason, the calibration process was repeated for several tip-to-sample distances using the laser measurement device to fix the distance.

**Figure 5:**
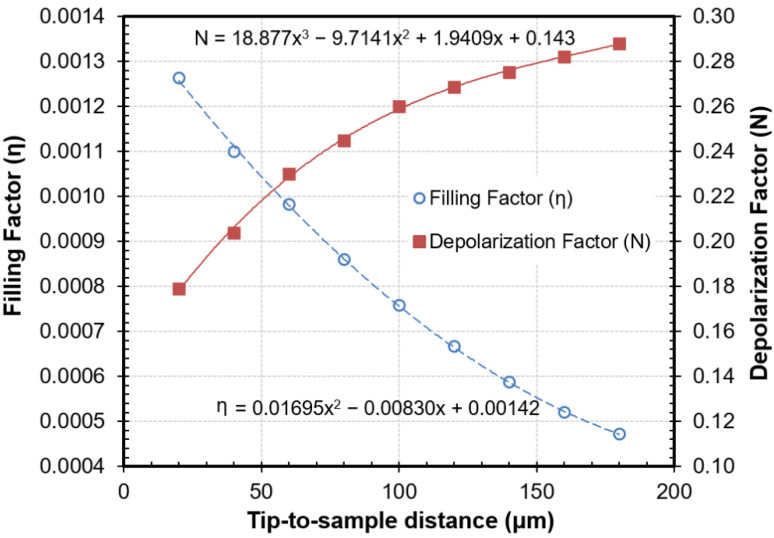
Calibration parameters, *η* and *N*, of the cavity perturbation method determined with reference samples as a function of the tip-to-sample distance (referred to as x in the regression equations).

Figure 5 shows the variation of the CPM *N* and *η* parameters as a function of the tip-to-sample distance. As illustrated, the two smooth curves were thoroughly adjusted with two polynomials of second and third degree, in order to find any value of these parameters that was in the vicinity of 100 μm. With the help of these curves, once the reading of the resonator and the laser had been recorded, the permittivity value of the MUT could be calculated in a straightforward manner for each position from (1) and (2).

## 4. Experimental Results

### 4.1. Dielectric Measurements

To assess the performance of the proposed NSMM measurement setup and the CPM calibration, the permittivity values of six reference specimens were determined from resonance measurements at different tip-to-sample distances around 100 μm.

The materials employed to undertake this assessment were the set of dielectric materials whose resonant frequencies were measured in Section 3.1, covering a broad range of permittivity values. The dielectric materials were placed on the base of the microscope and centered on the axis of the coaxial probe. With the aid of the laser, the Z-axis was moved from the soft-contact point to the studied tip-to-sample distances. From the displayed distance, the N and η values of each measurement were extrapolated from Figure 5, and the permittivity values were determined by means of the CPM technique from the resonance parameters provided by the reflectometer. All measurements were performed at 23 °C.

Table 1 shows the results of dielectric constant of all considered samples. The loss factor results were omitted from this study because of the weak sensitivity of the quality factor achieved with these materials for this probe size and tip-to-sample distances. To evaluate the accuracy of the results, all dielectric materials were also measured, as references, in a partially loaded cylindrical cavity [42] to evaluate the accuracy of the results.

The dielectric constant results agreed very well with the reference values in that all the materials and tip-to-sample distances displayed similar deviations. For short tip-to-sample distances, miss-positioning of the vertical axis caused deviations in the resonant frequency that were compensated for with the higher frequency shifts. On the other hand, for longer tip-to-sample distances, resonance deviations, due to inaccuracies when determining the tip-to-sample distances, became less relevant, but the influence on the dielectric constant increased because of the reduced frequency shifts. The error achieved for each material, defined as the absolute difference between the measured mean value and the reference value (|Δ*ε′*/*ε′*|%), was below 2%, and the standard deviation was mostly below 3%. Withal, each individual deviation for each measurement was slightly higher, with all the results falling within 5% of the reference value. This uncertainty is comparable to the uncertainty reported in [34], which achieved accuracies below 10% with a sophisticated tip-to-sample control system and a full-featured VNA as a measurement device.

### 4.2. Watermark Dielectric Maps

The developed NSMM device was used to scan the variations of permittivity of banknote watermarks, one of the most readily recognized security features available to the user for the authentication of banknotes or other public documents, such as passports. In this study, we utilized a second series 20 EUR banknote, which contains a watermark depicting a portrait of Europa, a figure from Greek mythology, as well as a Gothic-style window and the value numeral of the banknote.

**Figure 6:**
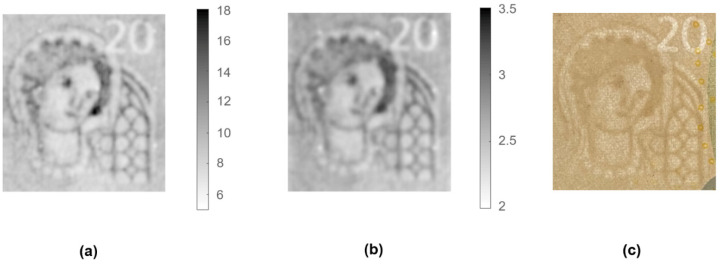
Images of the watermark included in a 20 EUR banknote: (**a**) dielectric image measured with the metallic base, (**b**) dielectric image measured with the PVC base, and (**c**) picture of the measured watermark using transmitted light.

The banknote was placed on the metallic structure of the XY-stage equipped with a suction vacuum table, which fixed the document during the scanning. With the aid of the displacement laser, the tip of the probe was placed at a distance of 100 μm from the surface of the banknote. The XY-stage covered an area of 33 mm × 29 mm with a step of 100 μm. The displacement laser information was employed to retrieve the appropriate CPM parameters for the tip-to-sample distance variations during the scan. Figure 6a shows the dielectric image of the watermark area obtained through the CPM calculation of the resonance parameter variations of the scan. For comparison, Figure 6c also shows a picture of the measured watermark using transmitted light. To have a numeric reference, dielectric properties of paper money were measured with a split-post dielectric resonator [43], achieving the value: 2.89 ± 0.2 − j0.3 ± 0.02.

The scanned dielectric image allows the watermark to be clearly recognized if compared with the image recovered through optical means. The measured dielectric constant values (from 6 to 18) are higher than the reference value, which indicates that the microwave signal penetrates through the whole banknote thickness and is then reflected by the metal base, influencing measured resonance parameters.

In order to avoid these reflections, the metal base below the banknote was substituted by a PVC (polyvinyl chloride) perforated table, whose permittivity—3.09 ± 0.06 − j0.023 ± 0.002—was fairly similar to the dielectric properties of the bulk banknotes. Figure 6b shows the dielectric constant response of the NSMM device for the watermark and setup described above, except for the material of the XY-base. The watermark pattern was recovered accurately again, albeit exhibiting a slightly lower sharpness. The dielectric constant values ranged from 2 for the lightest parts of the watermark (lower fiber densities) to 3.5 for the darkest parts (higher fiber densities). These results are in line with those measured with the split-post dielectric resonator and other values reported in the literature [44,45].

The penetration of microwaves was able to reproduce the density changes inside the papers and therefore NSMM devices could offer an alternative technology to identify anti-counterfeiting watermarks on banknotes. This capability could predict the detection of watermarks even when hidden behind appended layers. Next, we examined two different options to detect optically opaque markers.

**Figure 7:**
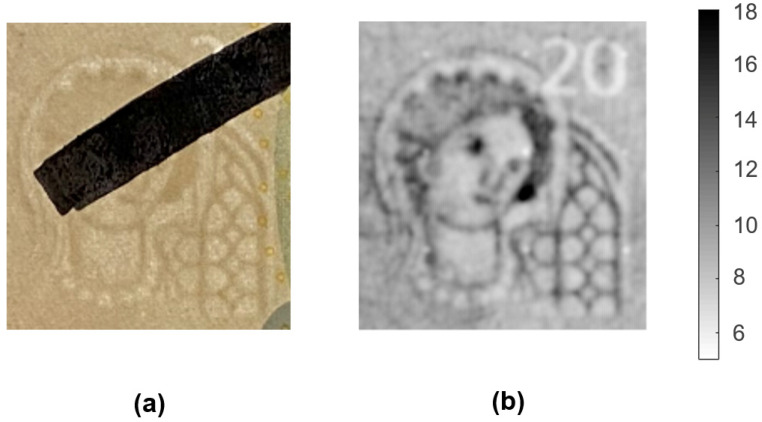
Images of the watermark included in a 20 EUR banknote partially covered with a black mark: (**a**) picture of the studied watermark using transmitted light and (**b**) dielectric image obtained with the metallic base.

In the first experiment, the watermark was partially hidden by a black mark drawn with a felt pen to study the influence of a thin ink coating over the watermark, as depicted in Figure 7a. As in the previous experiments, the sample was fixed by means of a vacuum suction system on the XY-table at a distance of 100 μm below the tip of the probe, collecting the microwave response over a 33 mm × 29 mm area. In these trials, we employed the metallic base of the NSMM. Although the values retrieved with the metal arrangement provided an effective dielectric map, the wider response of this setup served to increase its robustness for this specific application.

The obtained response, shown in Figure 7b, was very similar to the image shown in Figure 6a, with dielectric values ranging from 5 to 18. The mark made on the watermark did not cause any appreciable effect on the permittivity maps, demonstrating that the penetration of the microwave energy was not disturbed by such a thin dielectric coating.

Considering the previous results, in the second experiment, we employed a metallic cover to hide the watermark and complicate any reading by optical means. The mask consisted of a piece of a paper sheet (20 g/m^2^) painted with a layer of silver conductive paint (123-9911, RS Components, Corby, UK). As long as the thickness of the metallic layer was less than the skin depth of the conductive paint at the working frequency (~35 μm) [9], microwave energy could propagate through the mask and detect the changes in the density of the banknote watermark placed beneath it. As in the previous experiments, the tip-to-sample distance was 100 μm, but, in this case, this distance was measured from the surface of the metallic cover, and thus the separation between the watermark and the tip of the probe was slightly higher.

**Figure 8:**
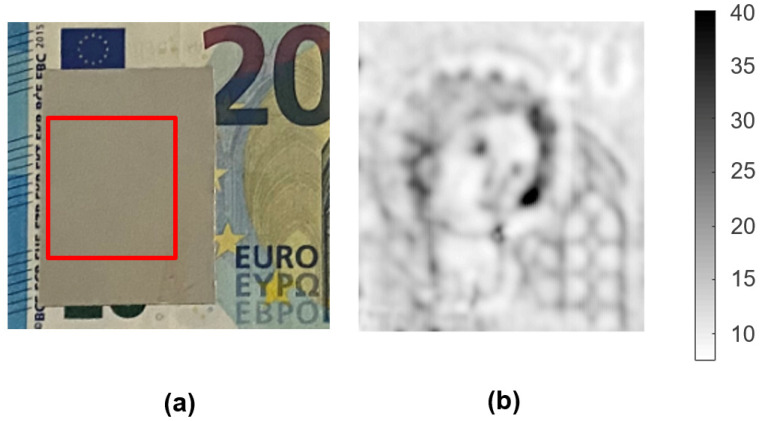
Images of the watermark included in a 20 EUR banknote partially covered with a metallic mask: (**a**) picture of the measured watermark and (**b**) dielectric image obtained with the metallic base.

The image recovered from the concealed sample (Figure 8b) displayed once again a reliable overall quality, with permittivity values ranging from 8 to 40. The influence of the metallic ink pushed up the dielectric values and extended the dielectric range, due to the saturation in the dielectric response as permittivity increased, as mentioned in Section 3.1. However, we observed a reduced spatial resolution due to the attenuation of the microwave signals passing through the metal film and the larger tip-to-sample distance. The non-uniformity of the metallic ink mask could have an influence as well.

### 4.3. Dielectric Scans at Single Frequency

The permittivity maps, represented in previous sections, required a frequency sweep around the resonant peak of the cavity to measure the reflection coefficient, calculate the *f_r_* and *Q*, and, eventually, to calculate the permittivity values. This process can last around 2 s per sweep, which makes the whole process very time consuming. However, surface scans can significantly reduce the overall scanning time by using the magnitude or phase of the reflection coefficient at a fixed frequency in the slope of the resonance. Although permittivity cannot be calculated with this working procedure, it could nevertheless still be valid for some sensing applications.

**Figure 9:**
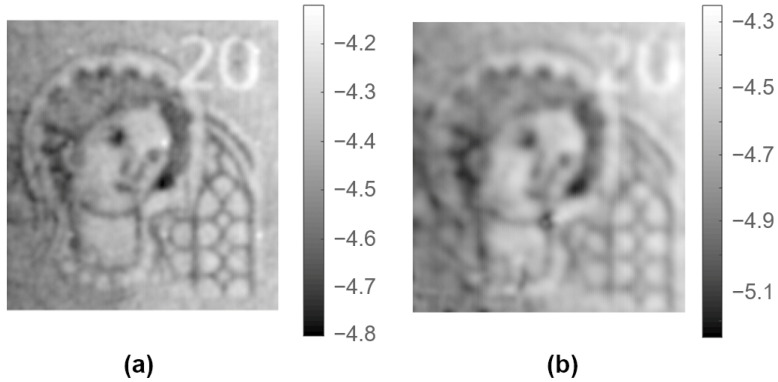
Images of the watermark included in a 20 EUR banknote using the magnitude (dB) of the reflection coefficient at a fixed frequency of 1.82 GHz and the metallic base; (**a**) watermark partially covered with a black mark and (**b**) partially covered with a metallic mask.

Figure 9a,b shows the images generated by the NSMM device of the watermarks represented in Figure 7a and Figure 8a, respectively, using the magnitude values of the reflection coefficient measured by the reflectometer at a single frequency (*f* = 1.82 GHz), located on the left side of the resonance peak. In this case, the laser was only employed to fix the central tip-to-sample distance without considering any variation during the scan.

The measurement image of the watermark covered with dielectric ink (Figure 9a) was very similar to the permittivity map created with the full frequency sweep (Figure 7a). The image had excellent overall quality, and, even at certain points in the darker areas, the image of the dielectric mark improved due to the higher slope of the resonance. It was also observed that the left part of the image had a darker region than the right one, due to a slight non-horizontality of the XY table. The measurement of the watermark covered with metallic ink yielded an image much like the dielectric response shown in Figure 8b with the same effects highlighted before.

## 5. Conclusions

In this work, we describe for the first time the use of microwave technology for the identification of markers on anti-counterfeiting applications. Microwave detection of banknote watermarks was conducted with a robust, portable, and standalone NSMM device at the micrometer scale, specially developed to determine permittivity maps of heterogeneous banknotes or paper documents around the frequency of 2 GHz.

The near-field microwave probe was designed as an open-ended coaxial resonator with a tapered inner conductor. An in-house microwave reflectometer was developed in the microscope, thus avoiding the need for a full-featured VNA and allowing for standalone functionality. A micrometer-resolution XY-table and a displacement laser for vertical positioning completed the measurement setup of the NSMM.

A novel methodology, combining the information of the displacement laser and a modification of the CPM theory with calibration coefficients for different tip-to-sample distances was proposed to determine dielectric property maps of banknote watermarks, avoiding the need for more advanced distance-following techniques. Future work will consider finite thickness of laminate materials. Accurate dielectric measurements of reference materials revealed relative errors below 5%, similar to the deviations obtained with much more complex implementations.

The NSMM device was employed to measure the permittivity map of the watermark of a second series 20 EUR banknote under different conditions. The penetration of the microwaves was able to reproduce the density changes inside the papers, offering a practical alternative for the detection of anti-counterfeiting watermarks on banknotes. In addition, the capability of microwave energy to detect markers behind dielectric or even metallic layers was demonstrated, which opens new possibilities to develop more advanced security features such as watermarks concealed behind optically opaque elements, undetectable through traditional optical procedures. The single frequency approach also provided high-quality images with shorter measurement times, and thus highlights the convenience of this procedure for sensing applications.

The developed NSMM could also be employed as a fast and standalone tool to obtain permittivity maps of planar materials in a broad range of sensing applications.

## Figures and Tables

**Table 1:** Dielectric property results of reference materials and associated standard deviations.

	Tip-to-Sample Distance (μm)												
Material	20	40	60	80	100	120	140	160	180	$\varepsilon_{mean}^{\prime }\pm StdDev$	StdDev%	$\varepsilon_{ref}^{\prime }$	$\left|\frac{\Delta \varepsilon^{\prime }}{\varepsilon^{\prime }}\right|\%$
Air	1.00	1.00	1.00	1.00	1.00	1.00	1.00	1.00	1.00	1.00 ± 0.00	0.00	1.00	0.00
Rexolite	2.52	2.48	2.50	2.52	2.53	2.53	2.54	2.54	2.51	2.52 ± 0.02	0.83	2.53 ± 0.05	0.46
PVC	3.11	3.07	2.98	3.01	3.02	3.03	3.05	3.05	3.01	3.04 ± 0.04	1.30	3.09 ± 0.06	1.70
Macor	5.40	5.51	5.69	5.82	5.79	5.88	5.93	5.92	5.67	5.73 ± 0.18	3.23	5.68 ± 0.11	0.96
Alumina	9.11	9.31	8.59	8.86	9.13	9.27	8.96	8.73	8.77	8.97 ± 0.25	2.82	8.94 ± 0.18	0.33
Temex E41030	29.43	27.80	29.52	28.43	29.34	27.70	29.04	29.07	27.82	28.68 ± 0.75	2.66	28.28 ± 0.56	1.43
Temex E5980	67.10	68.80	67.90	67.43	68.19	66.30	67.62	67.14	66.02	67.39 ± 0.88	1.30	67.25 ± 1.34	0.21
